# Alpha-lipoic acid alleviates oxidative stress and brain damage in patients with sevoflurane anesthesia

**DOI:** 10.3389/fphar.2025.1572156

**Published:** 2025-03-20

**Authors:** Kailun Gao, Ying Wu, Yan Zhang, Pei Dang, Huanjia Xue, Teng Li, Meiyan Zhou, Liwei Wang, Yangzi Zhu

**Affiliations:** ^1^ Department of Anesthesiology, Xuzhou Clinical College of Xuzhou Medical University, Xuzhou Central Hospital, Xuzhou, China; ^2^ Jiangsu Province Key Laboratory of Anesthesiology, Xuzhou Medical University, Xuzhou, China

**Keywords:** sevoflurane, alpha-lipoic acid, oxidative stress, brain damage, 8-OHdG, S100β

## Abstract

Sevoflurane, the most commonly used inhalational anesthetic, may negatively impact the brain by inducing oxidative stress. This study investigated the potential protective role of alpha-lipoic acid (ALA) in mitigating sevoflurane-induced oxidative stress and brain damage. A total of 155 patients undergoing sevoflurane anesthesia for liver resection surgery were randomly assigned to receive either ALA or a placebo. Perioperative internal jugular venous blood samples were collected to measure oxidative stress markers (8-OHdG, sORP, and cORP) and brain injury biomarkers (S100β and UCH-L1). Postoperative cognitive function was also evaluated. The results demonstrated that, compared to the placebo group, the ALA group exhibited a significant reduction in 8-OHdG levels by 0.007 nmol/L (95% CI, −0.011 to −0.003; P = 0.03) 24 h after surgery, accompanied by lower sORP levels and higher cORP levels. Furthermore, postoperative levels of S100β and UCH-L1 were significantly lower in the ALA group than in the placebo group (S100β, P = 0.02; UCH-L1, P = 0.03). Additionally, oxidative stress markers were significantly correlated with brain damage 24 h after surgery. Our findings suggest that ALA significantly reduces sevoflurane-induced oxidative stress and brain damage, while also improving postoperative cognitive function, indicating its potential neuroprotective effect.

**Clinical Trial Registration:** https://www.chictr.org.cn/, identifier ChiCTR2300077321.

## 1 Introduction

Sevoflurane, a volatile anesthetic widely used for its rapid induction and hemodynamic stability, is indispensable in prolonged surgical procedures (Jung et al., 2020; Orriach et al., 2013). Despite its clinical advantages, accumulating evidence suggests that sevoflurane may paradoxically exacerbate neuronal injury through oxidative stress, particularly during prolonged exposure (Liu et al., 2022; Senoner et al., 2021). Mechanistically, sevoflurane disrupts mitochondrial electron transport chain activity, leading to excessive reactive oxygen species (ROS) production, which overwhelms endogenous antioxidant defenses and induces oxidative DNA damage, lipid peroxidation, and protein misfolding (Niu et al., 2022; Tan et al., 2018). Notably, ROS overproduction activates pro-inflammatory pathways and promotes blood-brain barrier (BBB) dysfunction, contributing to postoperative cognitive dysfunction (POCD) and neuronal apoptosis (Zhang et al., 2018). These effects are further amplified in elderly patients or those undergoing prolonged anesthesia, highlighting an urgent need for adjunctive therapies to mitigate sevoflurane-associated neurotoxicity (Cao et al., 2023).

Recently, exogenous antioxidants have attracted attention in clinical settings because they are thought to regulate ROS production. Thus, they could serve as valuable interventions to enhance postoperative recovery (Stevens et al., 2019). Alpha-lipoic acid (ALA), which is a natural disulfide compound with strong antioxidant properties, has demonstrated significant protective effects against various oxidative stress-related conditions and diseases (Tripathi et al., 2023). Both ALA and its reduced form, namely, dihydrolipoic acid (DHLA), effectively scavenge ROS and promote endogenous antioxidant defenses by regenerating other antioxidants, such as vitamins C and E (Packer et al., 2001). Preclinical studies demonstrate that ALA crosses the BBB and exerts neuroprotection by activating the nuclear factor erythroid 2-related factor 2 (Nrf2) pathway, which upregulates antioxidant enzymes (Khan et al., 2022; Yang et al., 2017). In addition, ALA has been shown to reduce hippocampal oxidative damage, improve synaptic plasticity, and rescue cognitive impairment in animal models of ischemic stroke and Alzheimer’s disease (Fasipe et al., 2023). Despite the antioxidant mechanisms of ALA have been validated in preclinical studies, direct evidence supporting its application in perioperative neuroprotection remains lacking, particularly in the context of anesthesia.

This study aimed to investigate the effects of ALA on oxidative stress and brain injury in patients under sevoflurane anesthesia. We hypothesized that ALA would mitigate ROS-mediated DNA damage, restore redox homeostasis, and reduce biomarkers of brain damage. Our findings provide critical insights into ALA’s role as a perioperative neuroprotectant and its capacity to modulate anesthesia-related oxidative pathways.

## 2 Materials and methods

### 2.1 Study population

This study began in November 2023 and included patients who underwent liver resection under sevoflurane anesthesia, with recruitment continuing until November 2024. The inclusion criteria were as follows: at least 18 years of age, classification of as ASA I to III, and eligibility for liver resection with an expected operation time of at least 2 hours. The exclusion criteria included contraindications to sevoflurane; severe diseases affecting major organs (heart, liver, lungs, or kidneys); serious infectious; endocrine, hematologic, or neuropsychiatric disorders; history of smoking, excessive alcohol consumption, or substance misuse; and recent exposure to radiation or antioxidant medications within 1 week before surgery. All the participants provided written informed consent, which was submitted to the Clinical Trial Research Center for record-keeping. This study was approved by the Ethics Committee of Xuzhou Central Hospital (XZXY-LK-20231029-0178) and registered with the Chinese Clinical Trial Registry (ChiCTR2300077321).

### 2.2 Study procedure

**FIGURE 1:**
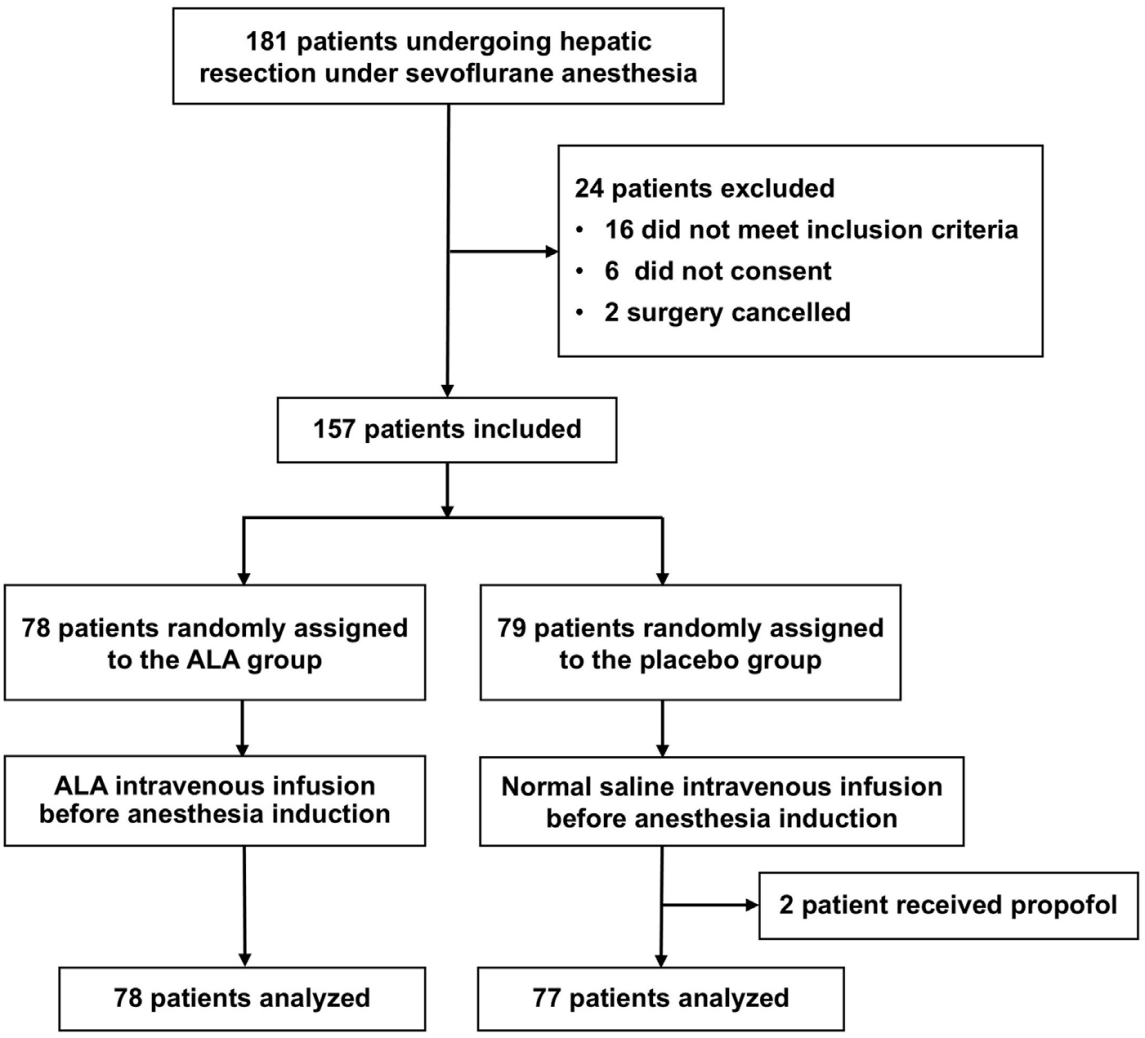
Flow diagram. A total of 157 patients were randomized, with 78 assigned to the ALA group and 79 to the placebo group. Two patients in the placebo group were excluded due to protocol deviations (intraoperative propofol use), resulting in 155 patients included in the final analysis.

Randomization was performed using a computer-generated randomization list with a block size of 6. Participants were randomly assigned to two groups: the ALA group and the placebo group. The ALA group received intravenous administration of α-lipoic acid before anesthesia induction, while the placebo group received an equivalent volume of normal saline (Figure 1). Solutions for both groups were prepared by a third-party pharmacist to ensure identical appearance and volume. Both participants and researchers remained blinded to group assignments until study completion.

A 600 mg dose of α-lipoic acid (Alpha-Lipon 300 Stada^®^, Stadapharm GmbH, Germany) was dissolved in 250 mL of normal saline and administered intravenously over 30 min. The 600 mg dosage was selected based on its established safety profile across diverse populations and its clinical application as a standard regimen for diabetic neuropathy treatment (Fogacci et al., 2020). Previous studies have demonstrated that intravenous administration of 600 mg ALA mitigates ischemia-reperfusion injury during organ transplantation surgeries (Ambrosi et al., 2016). Pharmacokinetic and pharmacodynamic analyses indicate that ALA’s therapeutic efficacy is primarily dependent on achieving adequate peak plasma concentration and area under the concentration-time curve, rather than time to peak concentration, elimination half-life, or mean residence time (Maczurek et al., 2008). Consequently, a single high-dose bolus may optimize cerebral delivery of ALA compared to fractionated dosing. To align with these findings, our study employed a single preoperative intravenous bolus of 600 mg ALA administered prior to anesthesia induction.

### 2.3 Anesthesia and perioperative management

Upon arrival at the operating room, standard monitoring of vital signs was performed for all patients, followed by internal jugular vein catheterization prior to anesthesia induction. General anesthesia was induced using intravenous midazolam (0.06–0.1 mg/kg), etomidate (0.2–0.4 mg/kg), sufentanil (0.4–0.5 μg/kg), and cisatracurium (0.15–0.25 mg/kg). During the procedure, all patients received a continuous infusion of remifentanil at a rate of 6–12 μg/(kg·h) and inhaled sevoflurane at a concentration of 1.5%–2.5%. Additional doses of cisatracurium (0.1–0.2 mg/kg) were administered every 30 min intraoperatively. The depth of anesthesia was adjusted to maintain a bispectral index (BIS) value between 40 and 60. Mechanical ventilation was maintained in volume-controlled mode, with a tidal volume of 6–8 mL/kg, an oxygen flow of 2 L/min, and a respiratory rate set to maintain normocapnia. Major liver surgery was defined as the resection of >3 anatomical segments or >6 non-anatomical resections and ablations (Reddy et al., 2011).

### 2.4 Sample collection

Blood samples were collected from the internal jugular vein at three time points: before anesthesia induction (T1), 2 h after induction (T2), and 24 h after surgery (T3). Blood samples were collected into vacuum tubes and processed immediately. The serum was separated by centrifugation at 1200 × g for 10 min at 4°C and stored at −80°C until further analysis.

### 2.5 Oxidative stress biomarkers

8-Hydroxy-2′-deoxyguanosine (8-OHdG), the primary endpoint of this study, is a specific biomarker of oxidative DNA damage and plays a critical role in neurodegenerative diseases and brain injury (Chao et al., 2021; Coppedè and Migliore, 2015). Elevated levels of 8-OHdG have been observed in clinical studies of Alzheimer’s disease, Parkinson’s disease, and other conditions, with some studies proposing 8-OHdG as an early marker of neurodegenerative disorders (Peña-Bautista et al., 2019). Previous clinical research has also confirmed that increased serum 8-OHdG levels are significantly associated with declines in early cognitive function scores, suggesting its utility as a sensitive indicator for evaluating the efficacy of antioxidant therapies (Cao and Chen, 2020; Liu et al., 2017). To enhance the specificity and accuracy of measurements, this study employed liquid chromatography-tandem mass spectrometry (LC-MS/MS), a method demonstrated to be more reliable than traditional ELISA techniques. The protocol was adapted from the study by Zou et al. (Zou et al., 2023). LC-MS/MS enables precise quantification of 8-OHdG, providing objective and quantifiable results throughout the perioperative period, thereby minimizing assessment bias.

The static oxidation‒reduction potential (sORP) and capacity oxidation‒reduction potential (cORP) were assessed with the RedoxSYS diagnostic system (Aytu Bioscience Inc.), following the protocol of Reiterer et al. (Reiterer et al., 2022). Measurements were conducted within 2 min of exposure to ambient air to avoid the effects of oxygen diffusion on the ORP. The sORP provides a measure of the current balance between all known and unknown oxidants and antioxidants, with a higher sORP (mV) indicating a greater level of oxidative stress (Reiterer et al., 2022). The cORP assesses a sample’s ability to withstand oxidative insults and is expressed in microcoulombs (μC). Typically, the higher the cORP is, the greater the antioxidant capacity (Bjugstad et al., 2016).

### 2.6 Brain damage biomarkers

S100β and UCH-L1 are used as biomarkers of brain damage. S100β is a calcium-binding protein that is found mainly in astrocytes and oligodendrocytes, and it serves as a sensitive biomarker of brain damage. Its levels often increase before clinical or neuroimaging changes are detected (Papuć and Rejdak, 2020). Elevated S100β levels are associated with blood‒brain barrier disruption and postoperative cognitive dysfunction (Yin et al., 2024). UCH-L1 is a protein that is highly expressed in neurons, with its tissue distribution almost exclusively limited to the brain (Kwon et al., 2005). It is commonly used as a biomarker for acute neuronal injury and central nervous system (CNS) damage (Wang et al., 2017).

Serum concentrations of S100β and UCH-L1 were quantified by ELISA (MSKBIO, Wuhan, China). All the assays were performed according to the manufacturer’s instructions and were carried out in a single batch to reduce intraassay variability.

### 2.7 Cognitive function assessment

Cognitive function was assessed with the MoCA on postoperative days 1, 3, and 7, and this assessment was conducted by the same physician for consistency. The MoCA is a validated cognitive screening tool consisting of 30 items that evaluate various cognitive domains, including executive function, language, orientation, memory, and visuospatial abilities (Danquah et al., 2024).

### 2.8 Statistical analysis

The results of the pre-test revealed that the average increase in 8-OHdG levels in the placebo group was 0.038 ± 0.016 ng/mL in the 24 h after surgery. Assuming a clinically significant reduction of 20%, with an α value of 5% and a power of 80%, and accounting for a 10% dropout rate, a total of 156 patients were recruited.

Statistical analyses were performed using SPSS version 23 and R-Studio version 4.3.1. Baseline demographic characteristics were summarized descriptively. Categorical variables were reported as frequencies and percentages, while continuous variables were expressed as means with standard deviations (SD) or as medians with interquartile ranges (IQR). Differences between groups were assessed using the unpaired t-test for normally distributed continuous variables, the Mann-Whitney U test for non-normally distributed continuous variables, and the chi-square test or Fisher’s exact test for categorical variables. A linear mixed-effects model was employed to evaluate the effects of ALA on longitudinal changes in trial outcomes. Fixed effects included treatment group, time, group × time interaction. Baseline covariates (age, sex, BMI, preoperative MMSE score, duration of anesthesia, total ischemic time and intraoperative bleeding) were incorporated to adjust for potential confounding factors. Random intercepts for individual participants were incorporated to address within-subject correlations across repeated measurements. Results for continuous variables were presented as least-squares means (LS-means) with 95% confidence intervals (CI). Pearson correlation analysis was used to examine the relationships between oxidative stress markers (8-OHdG, sORP, and cORP) and brain injury biomarkers (S100β and UCH-L1) at time point T3. Statistical significance was set at p < 0.05.

## 3 Results

Baseline demographic and clinical characteristics are summarized in Table 1. No significant differences were observed between groups.

**TABLE 1:** Baseline characteristics of patients.

Characteristic	ALA group(n = 78)	Placebo group(n = 77)	p-value
Age (yr)	50.7 ± 9.3	50.1 ± 9.9	0.696^(**)^
Male, n (%)	55 (70.5)	46 (59.7)	0.159^(†)^
BMI (kg m^-2^)	22.1 ± 2.0	21.7 ± 1.7	0.180^(**)^
ASA Classification, n (%)			0.692^(†)^
1	12 (15.4)	10 (13.0)	
2	51 (65.4)	48 (62.3)	
3	15 (19.2)	19 (24.7)	
Medical history, n (%)			
Hypertension	23 (29.5)	18 (23.4)	0.388^(†)^
Diabetes mellitus	8 (10.3)	12 (15.6)	0.322^(†)^
Coronary heart disease	15 (19.2)	12 (15.6)	0.548^(†)^
Chronic pulmonary disease	10 (12.8)	8 (10.4)	0.637^(†)^
Chronic kidney disease	4 (5.1)	5 (6.5)	0.746^(††)^
Stroke	6 (7.7)	3 (3.9)	0.507^(††)^
Preoperative MMSE	27.4 ± 2.4	27.1 ± 2.1	0.408^(**)^
Child-Pugh Score, n (%)			0.273^(†)^
A	69 (88.5)	72 (93.5)	
B	9 (11.5)	5 (6.5)	
Surgical approach, n (%)			0.584^(†)^
Open	60 (76.9)	62 (80.5)	
Laparoscopic	18 (23.1)	15 (19.5)	
Type of liver resection, n (%)			0.294^(†)^
Minor LR	28 (35.9)	34 (44.2)	
Major LR	50 (64.1)	43 (55.8)	
Duration of surgery (min)	278 (236–331)	249 (227–301)	0.692^(*)^
Duration of anesthesia (min)	326 (277–387)	303 (249–338)	0.853^(*)^
Total ischemic time (min)	61 (38–75)	52 (33–61)	0.495^(*)^
Intraoperative bleeding (mL)	391 (233–431)	339 (255–394)	0.665^(*)^
Intraoperative transfusion, n (%)	17 (21.8)	12 (15.6)	0.322^(†)^
ICU stay(day)	1 (1–2)	1 (1–1.8)	0.414^(*)^

BMI, body mass index; ASA, american society of anesthesiology; MMSE, Mini-Mental State Examination. Data are shown as means ± SD, median (IQR) or as n (%).

^(*)^Mann-Whitney test.

^(**)^ t-test.

^(†)^Chi-square test.

^(††)^Fisher’s exact test.

### 3.1 Oxidative stress biomarkers

**FIGURE 2:**
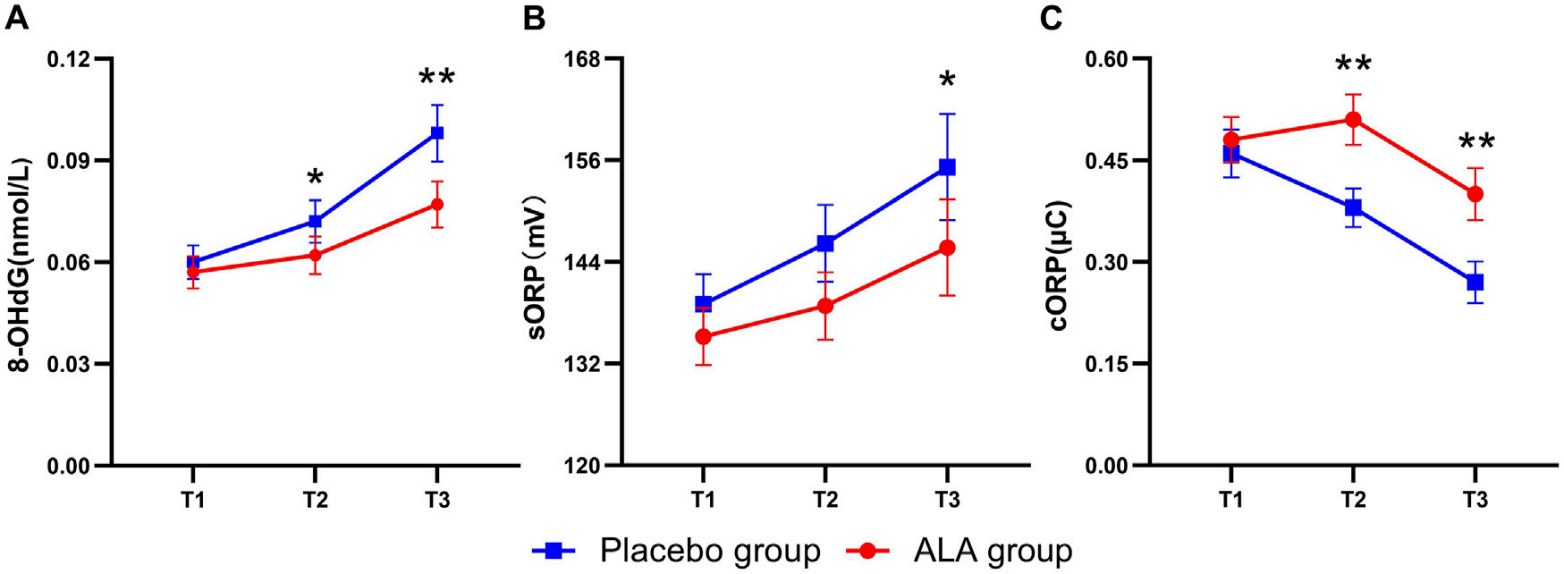
Dynamics of oxidative stress biomarker levels in the Placebo and ALA groups. Changes in 8-OHdG **(A)**, sORP **(B)**, and cORP **(C)** levels across T1 (before anesthesia induction), T2 (2 h after induction), and T3 (24 h after surgery) time points. Error bars indicate the standard error of the mean (SEM). Statistical significance is denoted as follows: *P < 0.05, **P < 0.01.

The dynamic analysis of oxidative stress markers (Figure 2A) revealed an increasing trend in 8-OHdG levels during and after surgery in both groups. However, the increase in the ALA group was significantly lower compared to the placebo group. Patients receiving ALA showed a reduction in intraoperative 8-OHdG levels by 0.007 nmol/L (95% CI, −0.011 to −0.003; P = 0.03) compared to the placebo group (Table 2). This effect persisted postoperatively, with a reduction of 0.015 nmol/L (95% CI, −0.024 to −0.006; P < 0.01), indicating that ALA significantly mitigated DNA oxidative damage induced by sevoflurane anesthesia. For sORP (Figure 2B), although levels increased during and after surgery, no significant difference was observed between the two groups intraoperatively. Postoperatively, however, sORP levels increased by 17.4 mV (95% CI, 12.2–22.6) in the placebo group compared to an increase of 10.5 mV (95% CI, 6.7–14.3) in the ALA group, resulting in a significant difference between groups (−6.9 mV; 95% CI, −13.3 to −0.5; P = 0.03, Table 2). In contrast, cORP (Figure 2C) demonstrated a downward trend throughout the perioperative period, possibly reflecting the depletion of antioxidant capacity. Notably, the ALA group showed an intraoperative increase of 0.03 μC (95% CI, −0.01–0.07), whereas the placebo group exhibited a decrease of −0.08 μC (95% CI, −0.13 to −0.03). Postoperatively, cORP levels in the ALA group were significantly higher compared to the placebo group, with an increase of 0.10 μC (95% CI, 0.01 to 0.19; P = 0.02). These findings suggest that ALA may help preserve antioxidant capacity and reduce perioperative depletion of antioxidant defenses.

**TABLE 2:** Effects of ALA on oxidative stress and brain damage.

Variable	Change from T1 (95% CIs)		Difference between groups (95% CI)
	ALA group (n = 78)	Placebo group (n = 77)	
8-OHdG (nmol/L)			
T2	0.005 (0.003–0.008)	0.012 (0.008–0.016)	−0.007 (-0.013 to -0.001)*
T3	0.020 (0.014–0.026)	0.035 (0.027–0.043)	−0.015 (-0.024 to -0.006)**
sORP (mV)			
T2	3.4 (−1.3–8.1)	7.7 (2.8–12.6)	−4.3 (−11.1 to 2.5)
T3	10.5 (6.7–14.3)	17.4 (12.2–22.6)	−6.9 (-13.3 to -0.5)*
cORP (μC)			
T2	0.03 (−0.01–0.07)	−0.08 (−0.13 to −0.03)	0.11 (0.05 to 0.17)*
T3	−0.08 (−0.13 to −0.03)	−0.18 (−0.25 to −0.11)	0.10 (0.01 to 0.19)*
S100β (pg/mL)			
T2	38.2 (22.5–53.9)	65.5 (45.9–85.1)	−27.3 (-50.4 to -4.2)*
T3	64.1 (49.5–78.7)	86.4 (70.8–101.9)	−22.3 (-43.6 to -1.0)*
UCH-L1 (ng/mL)			
T2	0.27 (−0.51–1.05)	0.74 (−0.16–1.64)	−0.47 (−1.66 to 0.72)
T3	1.94 (1.30–2.58)	3.45 (2.63–4.27)	−1.51 (-2.95 to -0.07)*

Analyses were conducted using a mixed-effects model with randomized treatment as a factor. Data are presented as least-squares means (95% CIs) for changes from baseline (T1). Bold values indicate statistically significant differences between groups (*P < 0.05, **P < 0.01). Analyses were adjusted for age, sex, BMI, anesthesia duration, total ischemic time, and intraoperative bleeding.

### 3.2 Brain damage biomarkers

**FIGURE 3:**
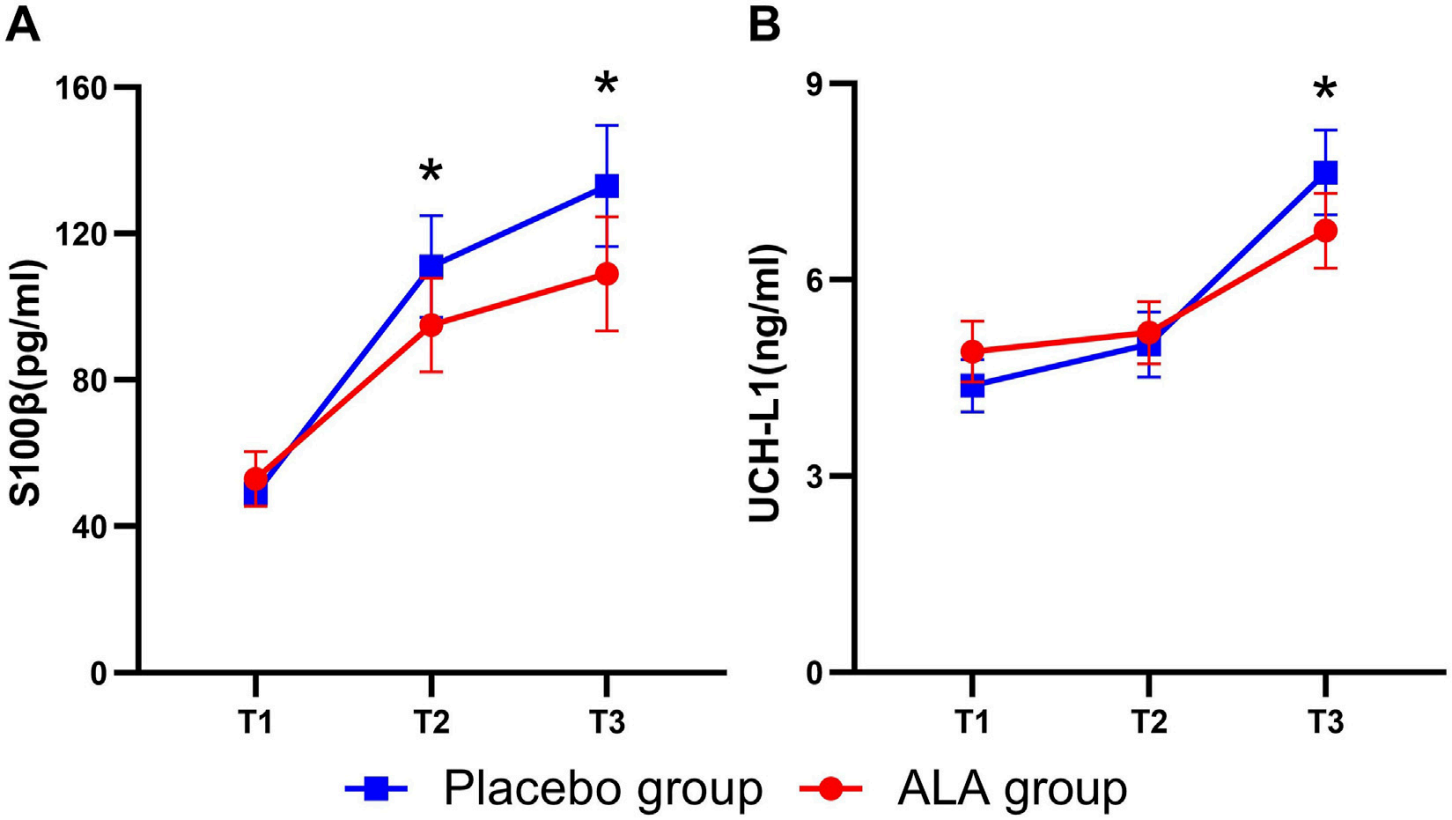
Dynamics of brain damage biomarker levels in the Placebo and ALA groups. Changes in S100β **(A)**, and UCH-L1 **(B)** levels across T1 (before anaesthesia induction), T2 (2 h after induction), and T3 (24 h after surgery) time points. Error bars indicate the standard error of the mean (SEM). Statistical significance is denoted as follows: *P < 0.05, **P < 0.01.

Figure 3 illustrates the dynamic changes in brain injury biomarkers (S100β and UCH-L1) in the placebo and ALA groups. Both biomarkers increased after anesthesia, with S100β showing a more pronounced rise intraoperatively, reflecting its high sensitivity to brain injury and rapid response in the early stages. ALA significantly reduced S100β levels 2 h after anesthesia (−27.3 pg/mL; 95% CI, −50.4 to −4.2; P = 0.01, Table 2), demonstrating early neuroprotective effects. Postoperatively, both S100β and UCH-L1 levels were significantly lower in the ALA group compared to the placebo group (S100β, P = 0.02; UCH-L1, P = 0.03). These findings suggest that ALA alleviates the elevation of S100β and UCH-L1 caused by sevoflurane anesthesia, thereby reducing perioperative neuronal injury.

### 3.3 Cognitive function assessment

On postoperative day 1, the MoCA scores in the ALA group were significantly higher than those in the Placebo group (25.0 vs. 24.2, p < 0.01, Table 3). Similarly, on postoperative day 3, the ALA group maintained significantly higher MoCA scores compared to the Placebo group (26.3 vs. 25.7, p = 0.03). However, by postoperative day 7, the difference in MoCA scores between the two groups was no longer significant (27.1 vs. 26.9, p = 0.3). These findings suggest that the ALA group demonstrated better cognitive recovery during the early postoperative period.

**TABLE 3:** Comparison of the MoCA scores between the two groups.

Group	1 day after surgery		3 d after surgery		7 d after surgery	
	x̄ ± SD	*P*	x̄ ± SD	*P*	x̄ ± SD	*P*
ALA group	25.0 ± 1.8	<0.01	26.3 ± 1.6	0.03	27.1 ± 1.2	0.3
Placebo group	24.2 ± 2.1		25.7 ± 1.7		26.9 ± 1.4	

The data are presented as mean ± standard deviation (x̄ ± SD). Between-group comparisons were performed using independent samples t-tests, with *P* < 0.05 indicating statistically significant differences.

**FIGURE 4:**
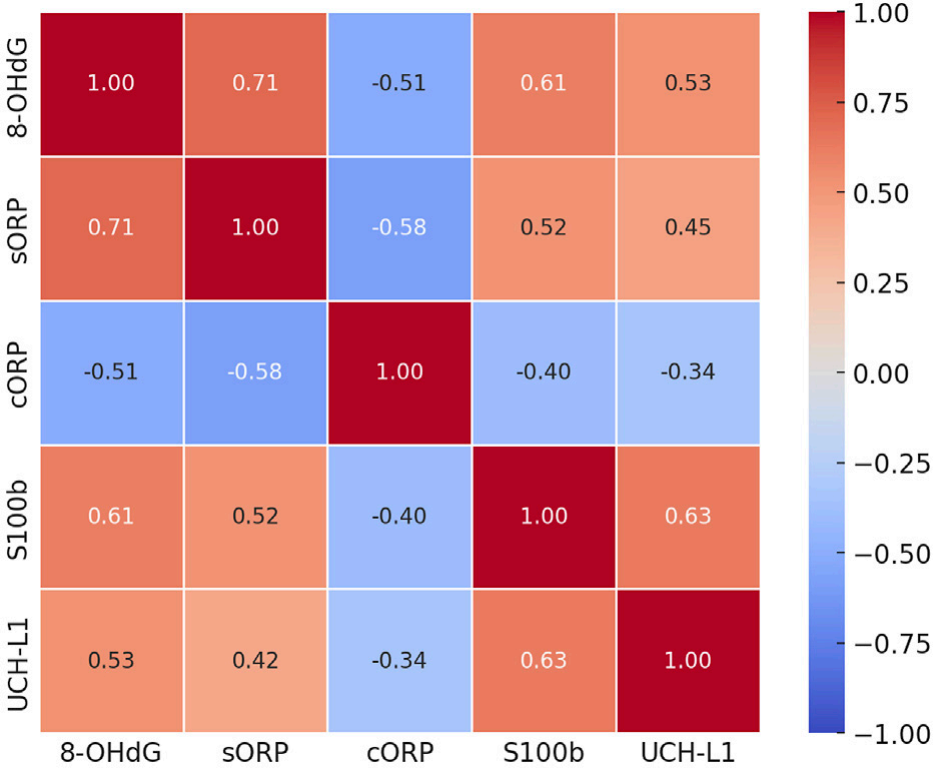
Correlation matrix of oxidative stress and brain damage markers 24 h after surgery. This heatmap shows Pearson correlation coefficients between oxidative stress markers (8-OHdG, sORP, and cORP) and brain damage markers (S100β and UCH-L1) at the T3 time point. Red indicates positive correlations, and blue indicates negative correlations. The values within each cell represent the correlation strength.

To investigate the potential relationships among biomarkers, we performed Pearson correlation analysis and visualized the results with a heatmap (Figure 4). The analysis revealed significant correlations between oxidative stress markers and brain damage markers at T3 (p < 0.05). Specifically, 8-OHdG showed a strong positive correlation with S100β (r = 0.61), indicating that as oxidative stress levels increase, brain damage marker levels increase correspondingly. Similarly, a significant positive correlation was observed between 8-OHdG and sORP (r = 0.71), highlighting their consistency in reflecting the oxidative stress status. In contrast, cORP, which is an indicator of antioxidant reserve capacity, was negatively correlated with 8-OHdG (r = −0.51), sORP(r = −0.58), S100β (r = −0.40), and UCH-L1 (r = −0.34), suggesting that reduced antioxidant capacity is associated with increased levels of oxidative stress and brain damage. The positive correlation between S100β and UCH-L1 (r = 0.63) suggests that these two markers may together reflect the severity of postoperative brain damage.

## 4 Discussion

In this randomized clinical trial, we found that preoperative administration of ALA effectively suppresses sevoflurane-induced oxidative stress and brain damage, improving postoperative cognitive function. Furthermore, higher levels of oxidative stress were significantly positively correlated with increased markers of brain damage. Our findings suggest that the antioxidant ALA holds promise as a potential neuroprotective agent in the perioperative setting. These results offer valuable insights for optimizing perioperative anesthetic strategies and mitigating brain injury.

The impact of general anesthetics on oxidative stress has been widely studied over the past few decades, but the specific effects of sevoflurane anesthesia on oxidative stress remain controversial (Stevens et al., 2019). In our study, sevoflurane anesthesia during liver resection significantly increased the 8-OHdG and sORP levels, while also reducing antioxidant capacity cORP. These findings are consistent with some publications in the literature. Previous studies have suggested that sevoflurane often exacerbates oxidative stress and DNA damage in patients undergoing major surgeries but poses lower risks in patients undergoing minor procedures, likely due to differences in the dosage and duration of exposure (Lee et al., 2015). In this study, all the participants underwent liver resection, which is a procedure that typically lasts more than 4 h; during this time, prolonged sevoflurane exposure may lead to excessive ROS production, exacerbating oxidative damage. Furthermore, preclinical evidence indicates that sevoflurane increases ROS levels in the brain, contributing to cognitive dysfunction (Yue et al., 2015). The accumulation of ROS not only triggers oxidative DNA damage but also may disrupt neuronal membranes and mitochondrial function, exacerbating neuroinflammation and brain injury (Piao et al., 2020; Yang and Yuan, 2018). Our results revealed significant increases in the S100β levels in the sevoflurane group, likely reflecting oxidative stress-related neuronal damage and increased blood‒brain barrier permeability (Yin et al., 2024). However, owing to its superior analgesic and muscle-relaxing properties and hemodynamic stability, sevoflurane remains a preferred choice for prolonged and complex surgeries (Brioni et al., 2017). Therefore, identifying effective measures to mitigate sevoflurane-induced oxidative stress and brain injury is critical for improving patient outcomes and safety.

In this context, ALA, which is a potent antioxidant with neuroprotective properties, has emerged as a promising therapeutic option to mitigate sevoflurane-induced oxidative stress and brain injury. ALA, which is a natural antioxidant, scavenges free radicals, modulates oxidative stress signaling pathways, and increases endogenous antioxidant defenses (Packer et al., 1995; Rochette et al., 2015). ALA has been demonstrated to be effective in repairing oxidative nerve damage and ameliorating diabetic peripheral neuropathy, and it is widely used in the prevention and treatment of complications of diabetes (Agathos et al., 2018). Furthermore, ALA readily crosses the blood‒brain barrier, which is a significant factor for CNS activities (Moraes et al., 2010). This characteristic positions were further supported by accumulating evidence from neurodegenerative disease research (Hager et al., 2007; Mishra et al., 2022). Notably, although preclinical studies suggest that DHLA may offer stronger antioxidant potential than ALA and can more effectively scavenge oxygen free radicals, its clinical translation is hindered by the lack of stable drug formulations and delivery protocols(Superti and Russo, 2024). Given these attributes, we selected ALA as an intervention to prevent sevoflurane-induced brain injury. To our knowledge, this is the first clinical study to explore the antioxidant and neuroprotective effects of ALA during anesthesia. Our results demonstrated that ALA significantly mitigated the increases in oxidative DNA damage and overall oxidative stress caused by sevoflurane while partially preserving antioxidant capacity. ALA also significantly reduced perioperative S100β levels and postoperative UCH-L1 levels, and improve cognitive function on postoperative days 1 and 3. However, the lack of a significant difference in MoCA scores at day 7 may reflect that the body’s natural recovery mechanisms partially masked the neuroprotective effect of ALA during later stages of postoperative recovery. Additionally, the transient benefits observed with a single ALA dose highlight its limited sustained efficacy, suggesting that repeated administration or prolonged treatment regimens may be required to amplify and prolong therapeutic gains. Future studies should include long-term follow-up trials to evaluate the sustained cognitive benefits of ALA and establish evidence-based dosing protocols, while also exploring DHLA-specific formulations or combinatorial strategies to optimize therapeutic efficacy.

This study also confirmed the relationship between oxidative stress and brain injury. Postoperative oxidative stress markers were significantly correlated with brain injury markers. These results suggest that elevated oxidative stress directly exacerbates postoperative neurological injury. The negative correlations of cORP with 8-OHdG and S100β further suggest that reduced antioxidant reserves are associated with increased oxidative stress and the severity of neurological injury. These findings reinforce the potential clinical utility of ALA as a reductive agent for perioperative neuroprotection. The brain’s high content of polyunsaturated fatty acids, its high oxygen consumption, and its limited antioxidant capacity render it particularly vulnerable to ROS (Evans, 1993; Singh et al., 2019). DNA damage caused by ROS can directly disrupt cellular function, leading to neuronal death and mitochondrial dysfunction (Chao et al., 2021). By scavenging free radicals and reinforcing the antioxidant barrier, ALA effectively reduces DNA oxidative damage, prevents neuronal apoptosis (Abdel-Aziz et al., 2021; Maciejczyk et al., 2022). However, perioperative oxidative stress is subject to multiple influencing factors, with the surgical intervention itself playing a significant role. In particular, liver resection involving ischemia-reperfusion procedures can lead to severe systemic oxidative stress, potentially amplifying the protective effects of ALA on brain injury. Furthermore, oxidative stress may be only one of the mechanisms underlying the neuroprotective effects of ALA. Future research should explore its specific mechanisms, including inflammatory pathways, to enhance our understanding of its neuroprotective effects.

This study has certain limitations. First, the sampling time points were limited, and the dynamic changes in some biomarkers may not have been completely captured. Although key time points, such as 2 h after anesthesia induction and 24 h after surgery, provide valuable data, future studies should optimize sampling schedules and use advanced techniques to better elucidate the dynamic changes in oxidative stress and brain injury associated with sevoflurane anesthesia. Second, the surgical procedure itself inevitably influences oxidative stress, which is a confounding factor that is difficult to completely eliminate in clinical research. To mitigate this, we included patients who underwent the same surgical procedure. Additionally, we measured biomarker levels in internal jugular venous blood, which primarily collects cerebral venous blood without contribution of blood from other organs, effectively reducing interference from systemic metabolism and clearance (Wolahan et al., 2019). Finally, this study was conducted at a single center with participants of East Asian descent undergoing liver resection, which may limit the generalizability of the findings. Future trials that include diverse ethnic groups and a range of surgical contexts are needed to validate the broader applicability of the observed results.

## 5 Conclusion

Our study indicates that, ALA effectively suppresses sevoflurane-induced oxidative stress, reduces brain damage, and improves postoperative cognitive function, highlighting its potential for perioperative neuroprotection. Overall, this study underscores the critical role of oxidative stress in sevoflurane-induced brain damage and suggests that ALA could be a promising therapeutic approach for mitigating such damage, providing a scientific basis for neuroprotection in perioperative patients.

## Data Availability

The original contributions presented in the study are included in the article/supplementary material, further inquiries can be directed to the corresponding authors.
